# A Novel Iterative CT Reconstruction Approach Based on FBP Algorithm

**DOI:** 10.1371/journal.pone.0138498

**Published:** 2015-09-29

**Authors:** Hongli Shi, Shuqian Luo, Zhi Yang, Geming Wu

**Affiliations:** School of Biomedical Engineering, Capital Medical University of China, Beijing, China, 100069; Zhejiang Univ, CHINA

## Abstract

The Filtered Back-Projection (FBP) algorithm and its modified versions are the most important techniques for CT (Computerized tomography) reconstruction, however, it may produce aliasing degradation in the reconstructed images due to projection discretization. The general iterative reconstruction (IR) algorithms suffer from their heavy calculation burden and other drawbacks. In this paper, an iterative FBP approach is proposed to reduce the aliasing degradation. In the approach, the image reconstructed by FBP algorithm is treated as the intermediate image and projected along the original projection directions to produce the reprojection data. The difference between the original and reprojection data is filtered by a special digital filter, and then is reconstructed by FBP to produce a correction term. The correction term is added to the intermediate image to update it. This procedure can be performed iteratively to improve the reconstruction performance gradually until certain stopping criterion is satisfied. Some simulations and tests on real data show the proposed approach is better than FBP algorithm or some IR algorithms in term of some general image criteria. The calculation burden is several times that of FBP, which is much less than that of general IR algorithms and acceptable in the most situations. Therefore, the proposed algorithm has the potential applications in practical CT systems.

## Introduction

Today the medical images provide very important health information in clinical diagnosis, in which X-ray CT image is one of most important modalities. The quality of CT images heavily depends upon the reconstruction algorithms, especially in the case that only less and less projection data is available in order to reduce the radiation dose. The current algorithms can be roughly divided into two categories: 1) analytical reconstruction algorithms, 2) iterative reconstruction (IR) algorithms. Some of IR algorithms, such as the algorithms based on dictionary learning or compressed sensing(CS) theory, have become the research focuses and have been used in a few special fields, which can solve some reconstruction problems where projection data is far from the requirement of Shannon theorem [1–6]. However, these algorithms suffer from heavy calculation burden, poor convergence speed and other drawbacks. For example, the iteration number may be several hundreds of thousands [5], and the average computational time of one iteration may be several seconds [7]. Generally, IR algorithms are based on some hypothetical conditions which are not always satisfied in practice. For example, the algorithm using total variation minimization may cause the details to be weakened or removed. On other hand, the analytical schemes, i.e. FBP algorithm and its modified versions such as FDK (Feldkamp-Davis-Kress) algorithm, are much simpler and faster [8–14]. They have been used in almost all the fields of straight ray tomography, such as X-ray CT and PET (Positron Emission Tomography) [15–17].

For the continuous systems, Radon and inverse Radon transforms using FBP algorithm are in a close form in the mathematics principle [10, 11, 13]. However, it necessarily produces non-negligible aliasing degradation when the projection data and FBP algorithm have to be discretized. Many approaches had been proposed to deal with this problem. As the description in [18], a multilevel back-projection method had been presented to improve the computational speed while a point-spread-function (PSF) convolution techniques had been proposed to reduce blurring in reconstruction. As a result, the image quality was similar with or superior to that using the classic FBP technique. In [19], the spline interpolation and ramp filtering had been combined to improve the classic FBP algorithm, by which the image quality could also be improved somewhat. In [20], a new filter has been designed to substitute the classic ramp filter to improve reconstruction performance.

In this paper, an iterative CT reconstruction approach based on FBP algorithm is proposed, which is designed to hold both advantages while reduce both disadvantages of analytical and IR algorithms. In the algorithm, the classic FBP is utilized to obtain the initial reconstructed image, which is treated as the *intermediate* image. It is then projected along the original projection directions to produce the *reprojection* vectors. The difference between the real and reprojection vector is filtered by a special digital filter to produce the corrected projection term, which is then performed the inverse Radon transform with same parameters to produce a correction term in image domain. By adding the correction term to the previous one, the new intermediate image is obtained. The digital filter is designed to make the new reprojection vectors approach to the real projection vectors as much as possible. This procedure can be performed iteratively until certain stoping criterion is achieved.

Generally, the projection vectors (together with projection angles) are all the information that we have obtained about the image to be reconstructed. If the reprojection data obtained using the proposed approach better resembles the real projection data than the one obtained using the classic FBP, it is reasonable to regard the proposed approach is better than the latter one.

The digital filter is very important for the proposed approach. In order to make the idea behind the design scheme for the filter clear, the reason that produces aliasing degradation by the discretizing process is analyzed at first. Then, a correction scheme is proposed.

## Methods

At first of this section, FBP algorithm is introduced in brief, and then the aliasing degradation caused by the discretizing process in FBP algorithm is analyzed. At last, an iterative scheme is proposed to reduce the aliasing degradation.

### 1. FBP algorithm

There are two projection modes for the general tomography: parallel beam and fan beam projection. Since a reconstruction problem for the latter can be easily transformed as a problem for the former, only the former is studied in this paper.

**Fig 1:**
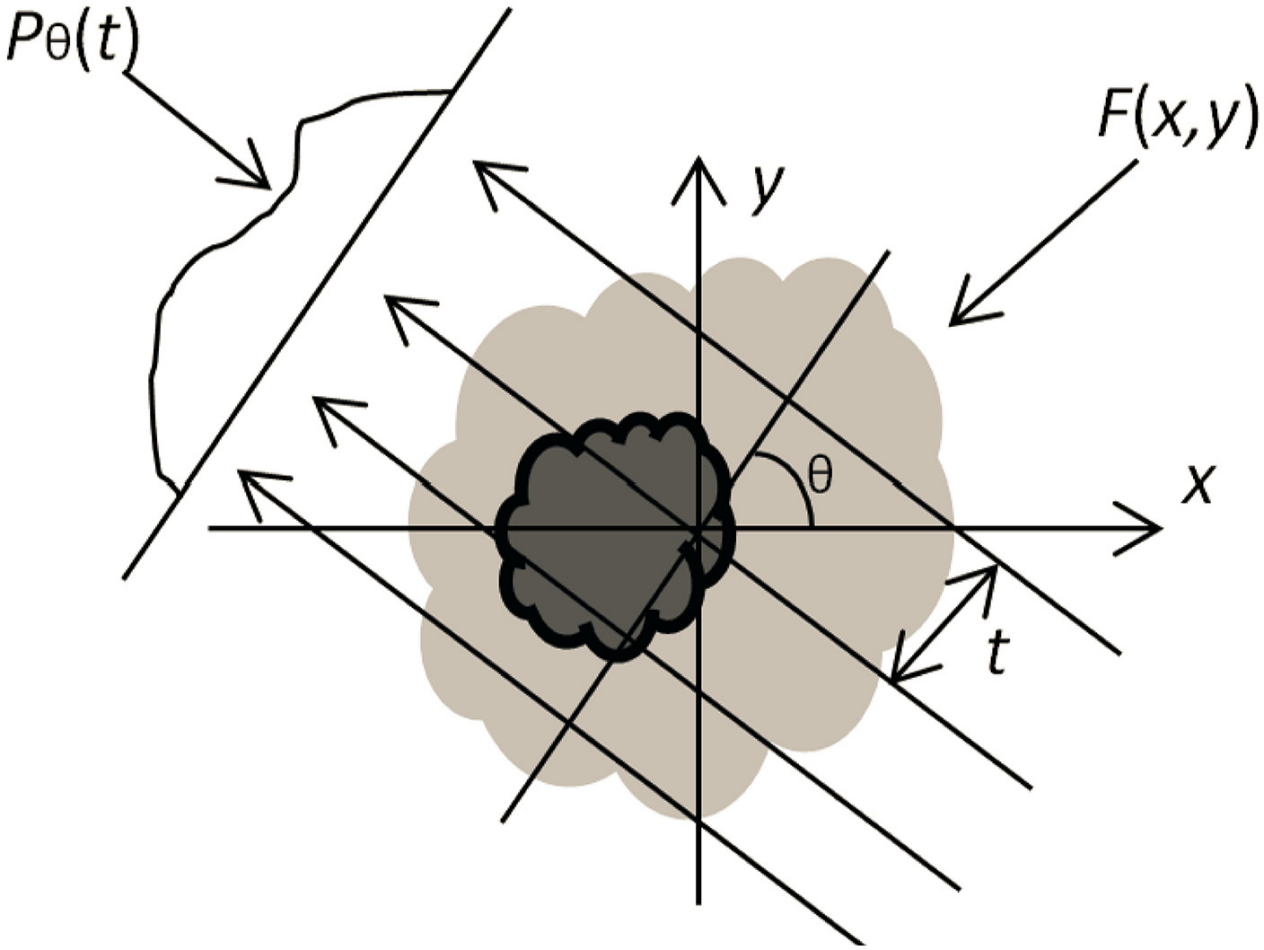
Parallel Projection: an object *f*(*x*, *y*) and its projection *p*_*θ*_(*t*) from angle *θ*.

The derivation of FBP algorithm for parallel beam tomography is rather simple and straightforward [10, 11]. The projection procedure can be instantiated in Fig 1. The Fourier slice theorem shows that the two-dimensional (2-D) Fourier transform (FT) at frequency sample (*ω* cos *θ*, *ω* sin *θ*), *F*(*ω* cos *θ*, *ω* sin *θ*), can be obtained by the one-dimensional (1-D) FT of projection vector at angle *θ* (at same time, the expression of beam lines in Cartesian coordinates is transformed as the expression in polar coordinates). Therefore, the 1-D inverse FT (IFT) can substitute 2-D IFT, by which the original image can be reconstructed, namely, the inverse Radon transform is achieved. The Jacobian matrix of Cartesian-polar transformation becomes as the ramp filter.

In the calculation, the projection data and reconstructed images have to be discretized. For a discrete projection vector, *p*_*θ*_*i*__(*t*), *t* ∈ [−*L*/2, ⋯, *L*/2] (denoted by *p*_*i*_(*t*) in the next), the discrete FT (DFT) and inverse DFT (IDFT) are employed to approximate FT and IFT, respectively. The FBP algorithm becomes [8]

$$\begin{array}{ccc} S_{i}\left(k\right) & = & \sum_{t=-L/2}^{L/2}p_{i}\left(t\right)\exp\left(-i2\pi \frac{kt}{N}\right),\;k\in \left[-N/2+1,\cdots ,N/2\right],\\ q_{i}\left(t\right) & = & \frac{1}{N}\sum_{k=-N/2+1}^{N/2}S_{i}\left(k\right)|\frac{k}{N}|\exp\left(i2\pi \frac{tk}{N}\right),\\ \hat{f}\left(n,m\right) & = & \frac{\pi }{K}\sum_{i=1}^{K}q_{i}\left(t\right)=\frac{\pi }{K}\sum_{i=1}^{K}q_{i}\left(\left\lfloor n\cos\theta_{i}+m\sin\theta_{i}\right\rceil \right), \end{array}$$ (1)

where $\hat{f}\left(n,m\right)$ denotes the reconstructed image; *L* is a positive even integer denotes the size of projection vectors; *N* is an even integer that is equal to or larger than the maximum size of the projection vectors at all directions; $|\frac{k}{N}|$ is the “ramp” filter in the frequency domain; ⌊*x*⌉ denotes the nearest integer of *x*; *θ*_*i*_, *i* ∈ [1, ⋯, *K*], denote the discretized scanning angle, and *K* is the number of the scanning angles. Generally, FFT (fast Fourier transform) is utilized to speed up reconstruction.

### 2. The aliasing degradation analysis

In this subsection, the implementation of FBP is analyzed in detail to show the cause of aliasing degradation. As shown in Eq (1), the implementation of FBP algorithm can be split into two steps: filtering and interpolating. The filtering can also be expressed in spatial domain as following after IDFT and some simplifications.

$$\begin{array}{ccc} q_{i}\left(t\right) & = & \frac{1}{N}\sum_{k=-N/2+1}^{N/2}[\sum_{l=-L/2}^{L/2}p_{i}\left(l\right)\exp\left(-i2\pi \frac{kl}{N}\right)]|\frac{k}{N}|\exp\left(i2\pi \frac{tk}{N}\right)\\ & = & \frac{1}{N}\sum_{l=-L/2}^{L/2}p_{i}\left(l\right)[\sum_{k=-N/2+1}^{N/2}\exp\left(-i2\pi \frac{kl-kt}{N}\right)|\frac{k}{N}|]\\ & = & \frac{1}{4}p_{i}\left(t\right)+\sum_{t\neq l}p_{i}\left(t\right)\beta_{N}\left(t-l\right) \end{array}$$ (2)

where

$$\begin{array}{c} \beta_{N}\left(t-l\right)=\frac{1}{N}(\sum_{k=0}^{N/2}\frac{k}{N}\exp\left(\frac{-i2\pi k(t-l)}{N}\right)-\sum_{k=-N/2+1}^{-1}\frac{k}{N}\exp\left(\frac{-i2\pi k(t-l)}{N}\right)) \end{array}$$

It is obvious that *β*_*N*_(*t* − *l*) become a constant when *N* is fixed. For example, when *N* = 64, *β*_54_(±1) ≈ −0.1014, *β*_64_(±2) ≈ 0, *β*_64_(±3) ≈ −0.0113; when *N* = 512, *β*_512_(±1) ≈ −0.1013, *β*_512_(±2) ≈ 0, *β*_512_(±3) ≈ −0.0112. In fact [9],

$$\begin{array}{c} \lim_{N\to \infty }\beta_{N}\left(t\right)=2\int_{0}^{1/2}x\cos\left(2t\pi x\right)dx=\{\begin{array}{cc} 1/4 & t=0,\;\;\;\;\;\;\;\;\;\;\;\\ 0 & t=\pm 2,\pm 4,\pm 6,\cdots ,\\ -\frac{1}{t^{2}\pi^{2}} & t=\pm 1,\pm 3,\pm 5,\cdots . \end{array} \end{array}$$

Let *β*(*t*) denote lim_*N* → ∞_ *β*_*N*_(*t*). When *N* is large enough such as *N* ≥ 128, *β*_*N*_(*t*) will be substituted by *β*(*t*) from now on, where the error caused by substitution will be very small and can be ignored. Since *β*(*t*) is symmetrical, the Eq (2) can also be expressed as the circular convolution of the original projection vectors and a kernel *h*_*n*_

$$\begin{array}{c} q_{i}\left(t\right)\approx p_{i}\left(t\right)⊗h_{n}\left(t\right) \end{array}$$ (3)

where ⊗ denotes the circular convolution operator, *h*_*n*_ = [*β*(2*n*+1), ⋯, *β*(3),0, *β*(1), $\frac{1}{4},\beta (1),0,\beta (3),0,\cdots ,\beta (2n+1)]$.

**Fig 2:**
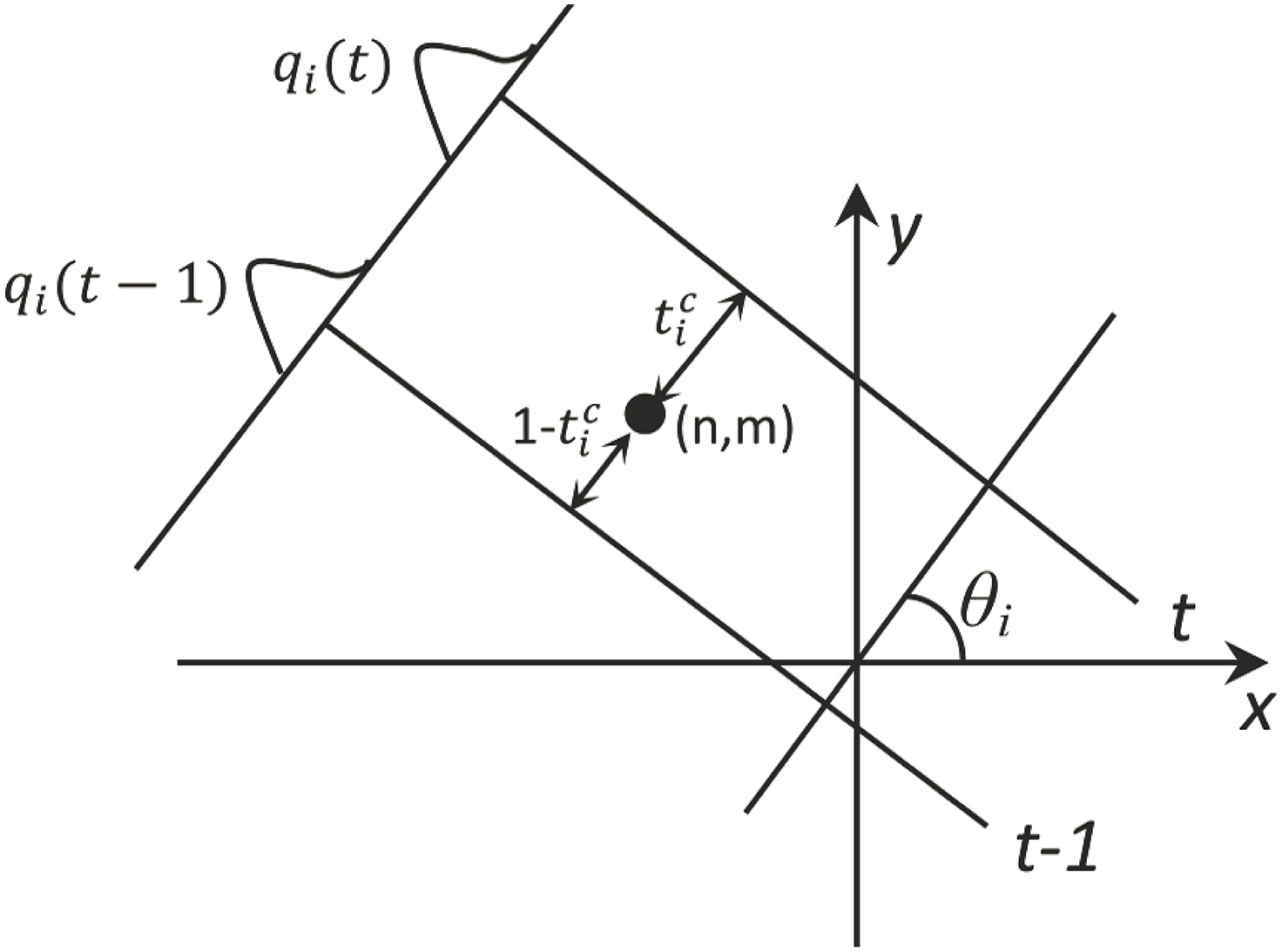
Reconstruction from the filtered projection: the linear interpolation procedure.

In the second step of FBP algorithm, for the filtered projection *q*_*i*_(*t*), the intensity of a pixel whose position (*n*, *m*) satisfy *t* − 1 < *n* cos *θ*_*i*_ + *m* sin *θ*_*i*_ < *t*+1 will be added with the weighted *q*_*i*_(*t*), which is rather similar with the discrete projection procedure. In that procedure, each pixel’s contribution is proportionally split into two nearest projection lines according to the distances between the projection lines and the pixel. The linear weighting coefficient is the common choice in this step, i.e., the intensity of point (*n*, *m*) will be increased by the linear interpolation of *q*_*i*_(*t*), *t* = −*L*/2, ⋯ *L*/2, which is shown in Fig 2. Finally, the reconstructed image is obtained by accumulating the contributions of all projection angles.

Now, suppose the reconstructed image is performed Radon transform along the identical angles again. The reprojection vector along *θ*_*i*_ with a distance *t* from the rotation center, ${\hat{p}}_{i}\left(t\right)$, can be expressed as

$$\begin{array}{c} {\hat{p}}_{i}\left(t\right)=\sum_{(n,m)\in S}s\left(t\right)\hat{f}\left(n,m\right) \end{array}$$

where *S* denotes the set of pixels that lies between *x* cos *θ*_*i*_ + *y* sin *θ*_*i*_ = *t* − 1 and *x* cos *θ*_*i*_ + *y* sin *θ*_*i*_ = *t* + 1; *s*(*t*) indicates the weight of a pixel contributes to the projection line ${\hat{p}}_{i}\left(t\right)$. Since each pixel’s contribution is proportionally split into the two projection lines that sandwich the pixel according to the distances between the projection lines and the pixel, *s*(*t*) is a linear function of *t*. By substituting $\hat{f}\left(n,m\right)$ with Eq (1), the reprojection vector can be expressed as following after some simplifications

$$\begin{array}{c} {\hat{p}}_{i}\left(t\right)=\sum_{(n,m)\in S}\sum_{j=1}^{K}\alpha_{i}\left(t\right)q_{i}\left(t\right) \end{array}$$ (4)

where *α*_*i*_(*t*) denotes the new coefficient, which is also the linear function of *t*. Consider Eq (3), the Eq (4) can further be expressed as following for briefness.

$$\begin{array}{c} {\hat{p}}_{i}\left(t\right)\approx \sum_{(n,m)\in S}\sum_{j=1}^{K}\alpha_{i}\left(t\right)p_{i}\left(t\right)⊗h_{n}\left(t\right)=\Theta_{i}\left(t\right)⊗h_{n}\left(t\right) \end{array}$$ (5)

where $\Theta_{i}(t)=\sum_{(n,m)\in S}\sum_{j=1}^{K}\alpha_{i}(t)p_{i}(t)$.

*Remark*: The Eq (5) is a summary conclusion about the original projection and reprojection vectors. It can also be comprehended in the following way. Since *q*_*i*_(*t*) ≈ *p*_*i*_(*t*) ⊗ *h*_*n*_(*t*), the interpolation in the reconstruction and the follow-up reprojection are all linear process when the linear interpolation mode is chosen in the reconstruction process, the reprojection vector can be expressed as the circular convolution of the ramp filter *h*_*n*_ and a term that is the linear combination of original projection vectors.

### 3. The correction scheme

**Fig 3:**
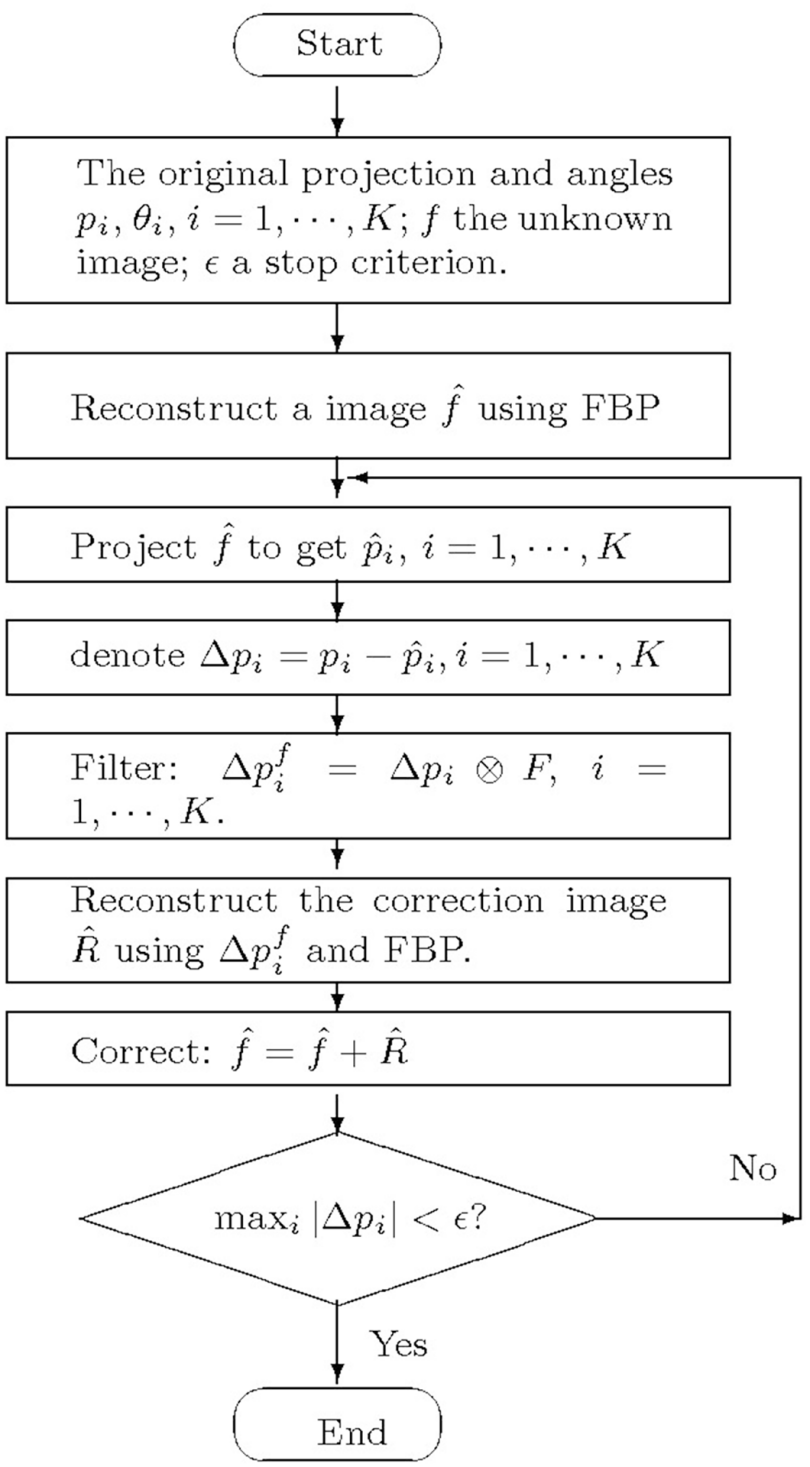
The flowchart of the iterative FBP algorithm.

The original projection vectors (and the corresponding projection angles) are the best knowledge we have got about the unknown object. The Eq (5) shows there is a distinct difference between an original projection vector and the corresponding reprojection one, which indicates the imperfection degree of reconstruction. We seek to take the advantage of the original projection data again in an iteration way to minimize the difference, in other words, improve the reconstructed image quality step by step. The proposed iterative scheme is shown as the flowchart in Fig 3.

The idea behind the processing flow is to make the reprojection vectors gradually approach the original projection vectors. First, obtain the initial reconstructed image $\hat{f}$ by FBP algorithm. $\hat{f}$ is then performed Radon transform using the identical setting to obtain the reprojection vectors ${\hat{p}}_{i}$. Second, calculate the difference between *p*_*i*_ and ${\hat{p}}_{i}$, $\Delta p_{i}=p_{i}-{\hat{p}}_{i}$, which is filtered to produce a filtered projection residual Δ^*f*^ *p*_*i*_ = Δ*p*_*i*_ ⊗ *F*. At last, an image correction term is obtained by Δ^*f*^ *p*_*i*_, which is add to $\hat{f}$ to update $\hat{f}$. The whole flow can be performed iteratively.

The design of the filter *F* becomes a very key issue. The filter should be designed to meet the following requirement

$$\begin{array}{c} \Theta \left(t\right)⊗h_{n}\left(t\right)+\left(\left[p\left(t\right)-\Theta \left(t\right)⊗h_{n}\left(t\right)\right]⊗F\left(t\right)\right)⊗h_{n}\left(t\right)\approx p\left(t\right) \end{array}$$ (6)

where *p*(*t*) denotes an original projection vector; Θ(*t*) ⊗ *h*_*n*_(*t*) denotes the corresponding reprojection vector; *p*(*t*) − Θ(*t*) ⊗ *h*_*n*_(*t*) denotes the projection residual, (*p*(*t*) − Θ(*t*) ⊗ *h*_*n*_(*t*)) ⊗ *F*(*t*) denotes the filtered version; ((*p*(*t*) − Θ(*t*) ⊗ *h*_*n*_(*t*)) ⊗ *F*(*t*)) ⊗ *h*_*n*_ denotes the correction term, which is added to Θ(*t*) ⊗ *h*_*n*_(*t*) to make the updated reprojection vector approach the original projection vector *p*(*t*). It requires

$$\begin{array}{c} h_{n}\left(t\right)⊗F\left(t\right)=\delta \left(t\right) \end{array}$$ (7)

where *δ* denotes the discrete Dirac delta function.

In the design, suppose *F*(*t*) is symmetrical and its length is identical with that of *h*_*n*_. For example, *h*_5_ = [*β*(−5),0, *β*(−3),0, *β*(−1),1/4, *β*(1),0, *β*(3),0, *β*(5)], *F* = [*x*_1_, *x*_2_, *x*_3_, *x*_4_, *x*_5_, *x*_6_, *x*_5_, *x*_4_, *x*_3_, *x*_2_, *x*_1_,]. *δ*(*t*) is

$$\begin{array}{c} \delta \left(t\right)=\{\begin{array}{cc} 1, & t=0\\ 0, & t\neq 0. \end{array} \end{array}$$

or *δ* = [0, 0, 0, 0, 0, 1, 0, 0, 0, 0, 0]

Now the design becomes rather simple. *F* will be the solution of the question

$$\begin{array}{c} \min_{F}{∥h_{n}⊗F-\delta ∥}_{2}^{2} \end{array}$$ (8)

For example, when *N* = 128, i.e., *h*_5_ = [−0.0041,0, −0.0113,0, −0.1013,0.25, −0.1013, 0, −0.0113, 0, −0.0041], then the normalized filter *F* = [0.0321, 0.0716, 0.1231, 0.1841, 0.3078,0.5625, 0.3078, 0.1841, 0.1231, 0.0716, 0.0321].

## Results

In this section, some numerical simulations and tests on real data are shown to demonstrate the performance of the proposed reconstruction algorithm together with the designed filter. All animal experiments and procedures carried out on the animals are approved by the animal welfare committee of Capital Medical University and the approval ID is SCXK-(Army) 2013-X-99.

**Fig 4:**
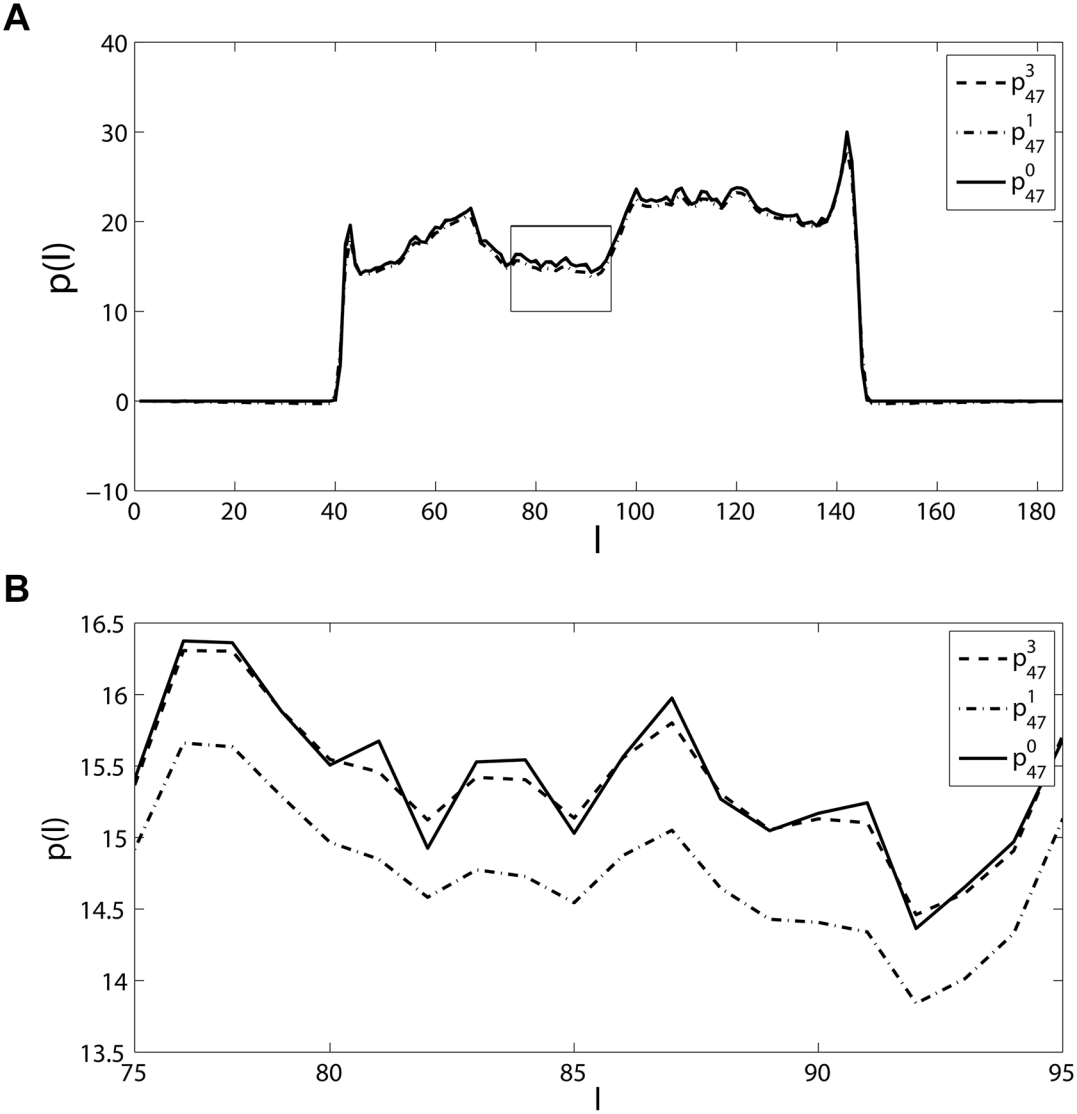
The original projection vector $p_{47}^{0}$, the reprojection vector, $p_{47}^{1}$, and the corrected projection vector, $p_{47}^{3}$, for the head phantom of size 128 × 128 and *θ* = 47°. (a) The whole vector, (b) the portion of the black in (a).

*Example 1*. This example is employed to show the filter designed can make the reprojection vectors approach the original projection vectors very well. The original image *I* selected is the head phantom generated by Matlab function *phantom* with 128 × 128 pixels. It is performed Radon transform using *radon* with the angle vector *θ* = [0°,1°, ⋯, 179°] to produce *p* ^0^. The reconstructed image $\hat{I}$ is obtained by *iradon* (where the classic FBP is utilized) using the linear interpolation mode. $\hat{I}$ is performed Radon transform again to get the reprojection vectors *p* ^1^. Then, calculate Δ*p* ^*f*^ = (*p* ^0^−*p* ^1^) ⊗ *F*, the correction image $\hat{R}$ and the corrected image $\hat{I}+\hat{R}$. Iteratively, a same Radon transform is performed to using the corrected image to produce new projection vectors, and so on. This procedure is iterated twice following the flowchart in Fig 3 (it is only a part of the proposed algorithm as the decision process is absent). Since they are matrixes (2d), only one vector of each *p* ^0^, *p* ^1^ and *p* ^3^ (the reprojection after twice iteration) is shown in Fig 4, whose angle is *θ* = 47°.

The results show that the difference between the corrected reprojection vector $p_{47}^{3}$ and $p_{47}^{0}$ is much smaller than that of the reprojection vector $p_{47}^{1}$ and $p_{47}^{0}$. In order to assess the efficiency of the designed filter, MSE (mean square error) is employed as the criterion. Let

$$\begin{array}{c} s_{1}=\frac{1}{LK}\sum_{i=1}^{K}∥p_{i}^{1}-p_{i}^{0}{∥}_{2}^{2},\;s_{2}=\frac{1}{LK}\sum_{i=1}^{K}{∥p_{i}^{3}-p_{i}^{0}∥}_{2}^{2} \end{array}$$ (9)

where $p_{i}^{0}$, $p_{i}^{1}$ and $p_{i}^{3}$ denote the original, reprojection and corrected projection vectors for the projection angle *θ*_*i*_; *L* is the size of a vector, and *K* denotes the number of projection angles. The results for two examples are shown in Table 1.

**Table 1:** MSE of the different projection and the original projection.

Image size *N*	128	1024
Angles interval	1°	0.3°
*s*_1_	0.2917	11.8474
*s*_2_	0.0322	0.0348

The results illustrate the proposed scheme together with the designed filter make the corrected reprojection vectors approach the true projection vectors very well.

**Fig 5:**
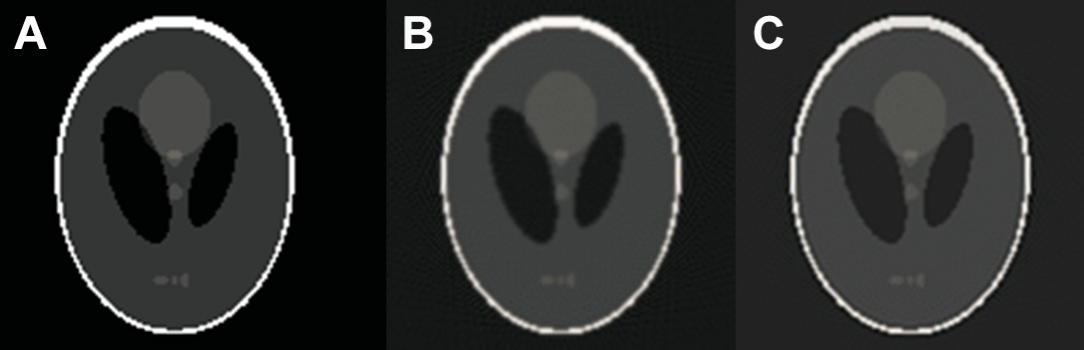
The original image and its reconstructed versions by different schemes. (a) The original image(128 × 128), (b) the reconstructed image using the classic FBP, (c) the reconstructed image using the iterative FBP.

*Example 2*. The original image *I* is also the phantom image of 128 × 128 pixels. The angle vector is selected *θ* = [0°,0.3°, ⋯, 179.7°], along which *I* is projected to produce the original projection *P*. It is reconstructed by the classic FBP and the proposed iterative FBP. The original image and its reconstructed versions by different schemes are shown in Fig 5.

As [20], three criteria, *MSE* (Mean Square Error), *UQI* (Universal Quality Index) and *MI* (mutual information), are employed to assess the efficiency of the proposed approach, which are defined as following [21, 22].

$$\begin{array}{c} MSE\left(I^{r},I^{o}\right)=\frac{1}{M}\sqrt{\sum_{k=0}^{M-1}{\left(I_{k}^{r}-I_{k}^{o}\right)}^{2}}, \end{array}$$

where $I_{k}^{r}$ and $I_{k}^{0}$ denote the pixels of the reconstructed image *I* ^*r*^ and reference image *I*°, respectively; *M* is the total number of pixels. *UQI* is defined as following

$$\begin{array}{c} UQI\left(I^{r},I^{o}\right)=\frac{2Cov(I^{r},I^{o})}{\sigma_{r}^{2}+\sigma_{0}^{2}}\frac{2{\bar{I}}^{r}{\bar{I}}^{o}}{{\left({\bar{I}}^{r}\right)}^{2}+{\left({\bar{I}}^{o}\right)}^{2}}, \end{array}$$

where $\bar{I}$ and *σ* ^2^ denote the image mean and variance, respectively; *Cov*(*I* ^*r*^, *I*°) denote the covariance of the reconstructed image *I* ^*r*^ and reference image *I*°. The mean, variance and covariance are defined as the following

$$\begin{array}{ccc} {\bar{I}}^{r} & = & \frac{1}{M}\sum_{k=0}^{M-1}I_{k}^{r},\;{\bar{I}}^{o}=\frac{1}{M}\sum_{k=0}^{M-1}I_{k}^{o}\\ \sigma_{r}^{2} & = & \frac{1}{M}\sum_{k=0}^{M-1}{\left(I_{k}^{r}-{\bar{I}}^{r}\right)}^{2},\;\sigma_{o}^{2}=\frac{1}{M}\sum_{k=0}^{M-1}{\left(I_{k}^{o}-{\bar{I}}^{o}\right)}^{2}\\ Cov(I^{r},I^{o}) & = & \frac{1}{M-1}\sum_{k=0}^{M-1}\left(I_{k}^{r}-{\bar{I}}^{r}\right)\left(I_{k}^{o}-{\bar{I}}^{o}\right). \end{array}$$

When the reconstructed image and reference image are interpreted as “stochastic processes”, *MI* is used for measuring their mutual dependence.

$$\begin{array}{c} MI\left(I^{r},I^{o}\right)=\sum_{k=0}^{N^{\prime }-1}\sum_{n=0}^{N^{\prime }-1}p\left(I_{k}^{r},I_{k}^{o}\right)\log(\frac{p(I_{k}^{r},I_{k}^{o})}{p\left(I_{k}^{r}\right)p\left(I_{k}^{o}\right)}), \end{array}$$

where $p(I_{k}^{r})$ and $p(I_{k}^{o})$ denote the marginal densities of *I* ^*r*^ and *I*°, respectively, which are calculated using the corresponding histograms; the joint density $p(I_{k}^{r},I_{k}^{o})$ is estimated from the joint histogram of *I* ^*r*^ and *I*°; *N*′ denotes the number of bins in the histogram.

*UQI* measures the pixel-to-pixel similarity between the reconstructed *I* ^*r*^ and reference image *I*°, while *MI* measures the histogram correlation between them. The closer to 1 the *UQI* value is, the more similar the two images are. Similarly, the higher the *MI* value is, the more similar the two images are. Since it is a simulation example, the original image is known and selected as the reference image.

The results in the form of the three criteria are showed in Table 2, in which the phantom images with different sizes and projection settings are employed. The results illustrate the proposed scheme have better reconstruction performance than the classic FBP algorithm.

**Table 2:** The performance evaluation in Example 2.

Image size	Angle interval	Algorithm	MSE	UQI	MI
128 × 128	1°	classic FBP	3.6864E-2	0.9543	0.9107
		Iterative FBP	1.1965E-2	0.9871	0.9205
128 × 128	0.3°	classic FBP	3.4766E-2	0.9545	0.9120
		Iterative FBP	1.271E-2	0.9883	0.9214
512 × 512	0.5°	classic FBP	0.4215E-2	0.9891	0.9502
		Iterative FBP	0.2170E-2	0.9940	0.9539
1024 × 1024	1°	classic FBP	7.747E-3	0.9789	0.9419
		Iterative FBP	3.278E-3	0.9848	0.9460
1024 × 1024	0.2°	classic FBP	1.644E-3	0.9945	0.9621
		Iterative FBP	0.5356E-3	0.9969	0.9629

*Example 3*. In order to compare the iterative FBP with the current iterative schemes in the terms of speed and accuracy, some reconstruction problems are implemented by SIRT (Simultaneous Iterative Reconstruction Technique), SART (Simultaneous Algebraic Reconstruction Technique) and MAP-EM (Maximum A Posteriori estimation Expectation Maximization) algorithms [23–25]. Unlike the iterative schemes such as the scheme based on minimization of total variation (TV) regularization or dictionary-learning, these algorithms do not make any artificial assumption on the image to be reconstructed. For the schemes based on TV minimization, there is an assumption that the TV of the underlying image is minimum. For the schemes based on dictionary-learning, the assumption is that the underlying image can be expressed by a linear combination of atoms, the high-dimensional vectors, in a dictionary, the vector set. SIRT, SART and MAP-EM schemes are identical with the proposed scheme in the regard, which depend only on the projection data rather than relying on a prior information or assumption. Thus, they are chosen to compare with the proposed scheme.

**Fig 6:**
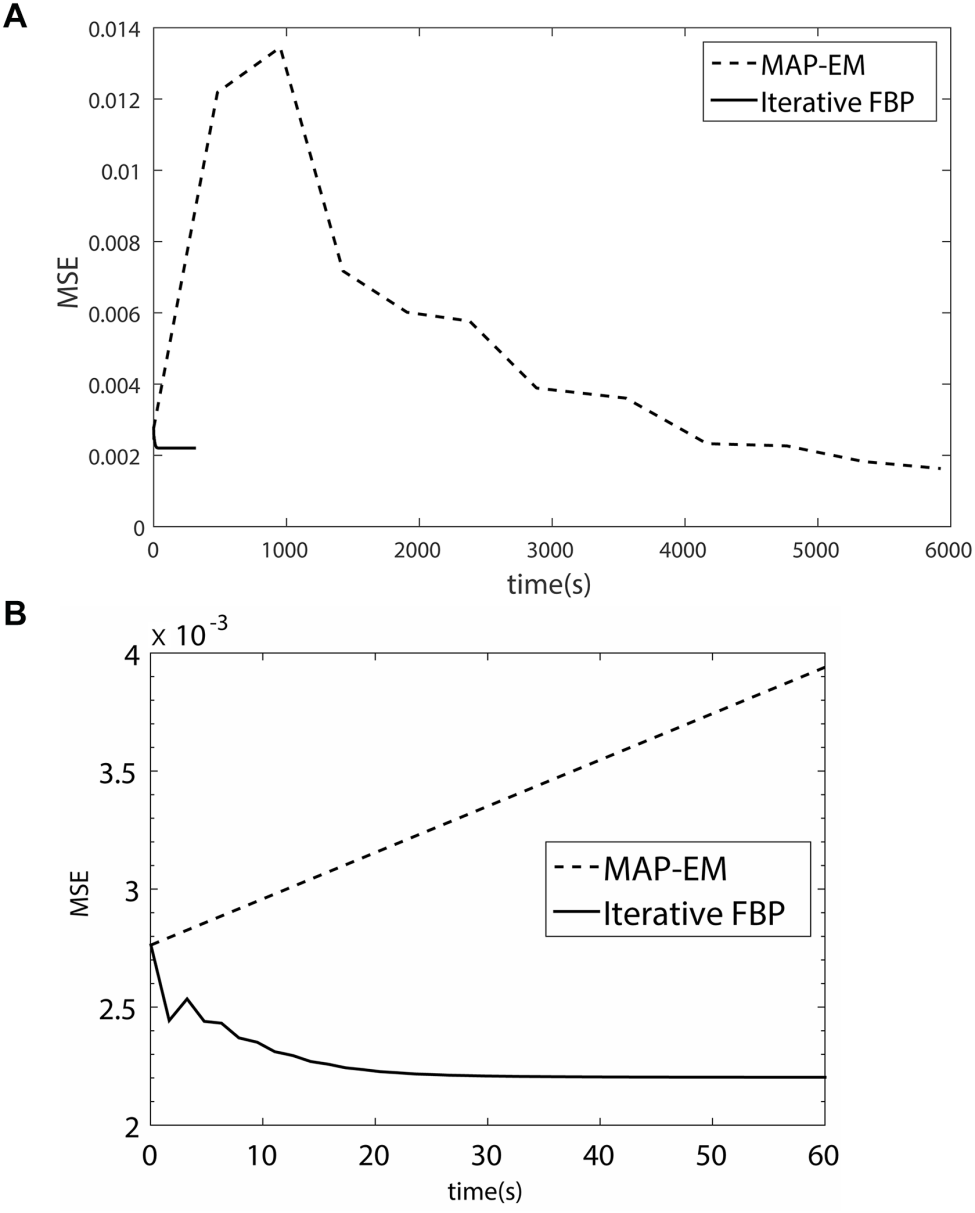
(a) The compare of MSE using MAP-EM and Iterative FBP. (b) is the portion of the first 60s of (a).

The head phantom with 512 × 512 pixels is selected as the original image, which is performed Radon transform with the angles *θ* = [0°,0.5°, ⋯, 179.5°] to produce original projection *p* ^0^. The image is reconstructed using the three schemes, and the three criteria, MSE, MI and UQI are employed to evaluated the reconstruction performance. For all the schemes, the image reconstructed using the classic FBP is chosen as the initial image of iteration. The results is showed in Table 3 and Fig 6.

**Table 3:** The compare of reconstruction performance between SIRT, SART, MAP-EM and Iterative FBP (the image size is 512 × 512 and the angle interval is 0.5°).

Algorithm	MSE	UQI	MI	performing time(s)
Iterative FBP	0.2170E-2	0.9942	0.9539	47
MAP-EM	0.2003E-2	0.9989	0.9595	5217
SIRT	0.1998E-2	0.9713	0.9417	58912
SART	0.2103E-2	0.9840	0.9539	67556

The results show that the iterative FBP algorithm has the similar reconstruction performance with SART, SIRT and MAP-EM schemes, and a much smaller operating time. We think the superiority of iterative FBP in the term of speed mainly roots in the fact that it does not employ the linear expression of projection procedure. The expression refers to a very huge matrix (see the Eq (1) or Eq (2) in [25] or in page 277 [8]) and will slow down the calculation, however, it is necessary for SIRT, SART and MAP-EM schemes. When the image size and the number of projection angle is large (as that in this example), the matrix becomes very huge and has to be split into many blocks. Every block has been saved and loaded at least one time, which spends many time.

**Fig 7:**
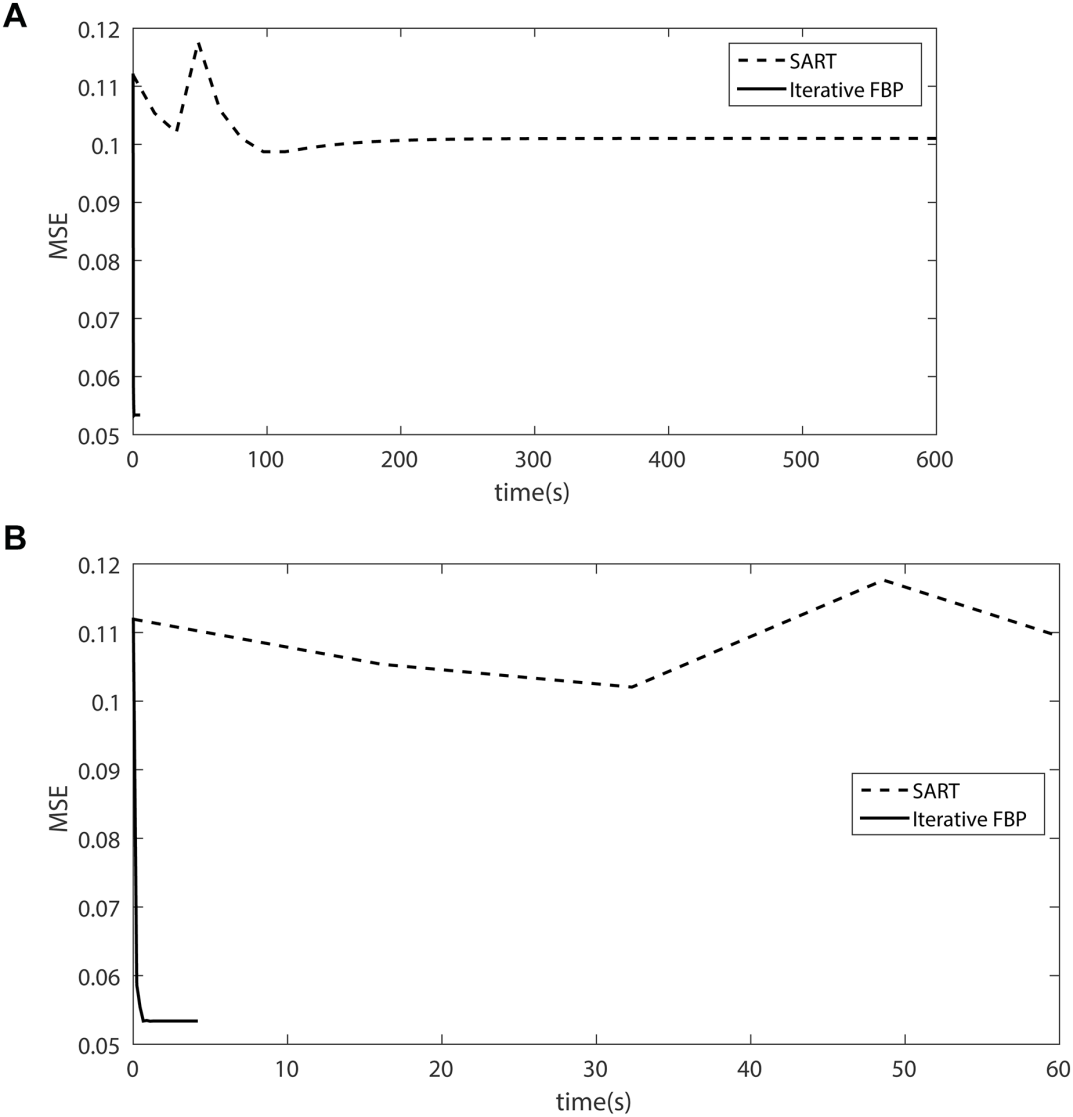
(a) The compare of MSE using SART and Iterative FBP. (b) is the portion of the first 60s of (a).

In order to analyze the robustness of iterative FBP, suppose the original projection is polluted by the white Gaussian noise, i.e.,

$$\begin{array}{c} p=p_{0}+\sigma Z \end{array}$$

where *p*_0_ denotes the original projection vectors, which is the Radon transform of the head phantom image of 256 × 256 pixels with the projection angles *θ* = [0°,1°, ⋯, 179°]; *Z* is the white Gaussian noise with same size as *p*_0_, whose mean is zero and variance is one; *σ* is the magnitude of noise, which is selected as 0.1max(*p*_0_). The image is reconstructed using the three schemes, SART, SIRT and MAP-EM, and Iterative FBP scheme. In all the schemes, the image that is reconstructed using the classic FBP is chosen as the initial image of iteration. The some results are showed in Fig 7.

The results show that the iterative FBP algorithm has the poor but acceptable reconstruction performance, while SART, SIRT and MAP-EM have very bad performance even after a rather long running time. SIRT and MAP-EM have worse tendency than SART with the iterative recurrence proceeds. We think the weaknesses mainly roots in the fact that they depend upon the linear expression of projection procedure, which make the reconstruction performance is very sensitive to the noise in the projection data.

**Fig 8:**
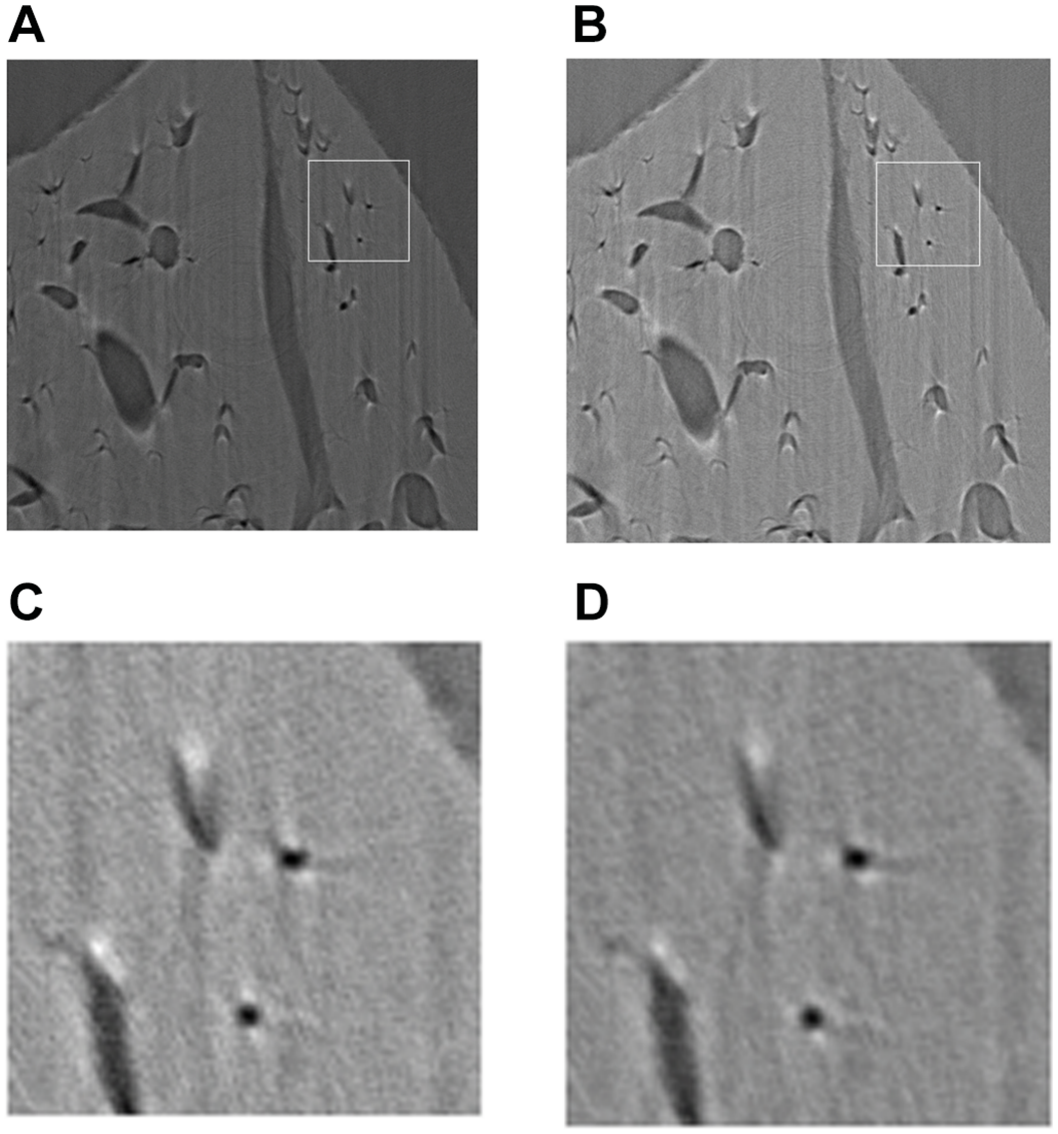
The images of mouse liver reconstructed using different schemes. (a) The image using the classic FBP, (b) using the iterative FBP, (c) and (d) are the portions of (a) and (b), respectively.

*Example 4*. In this example, the proposed algorithm is used in the practical application. The sample is a mouse liver, and the experiment is performed in the X-ray Imaging and Biomedical Application Beamline station (BL13W1) at the SSRF(Shanghai Synchrotron Radiation Facility, China). All animal experiments and procedures carried out on the animals are approved by the animal welfare committee of Capital Medical University and the approval ID is SCXK-(Army) 2013-X-99. The setup can ensure x-ray beam to be near parallel. The energy is about 21 keV. A CCD camera (the size of pixel is 13*μm* × 13*μm*) is used as the detector, comprising 2,588 × 458 pixels. During scanning, the sample is rotated on a turntable around its cylindrical axis by 180° at step of 0.4411°. The rotation speed is about 0.25°/*s* and the exposure time was 11 millisecond. Before and after scan, the background images (the sample is absent) and dark field images (the x-ray beam is closed) are recorded for preprocessing. The background images are used for normalization, the dark field images are used to reduce the noise of various kinds(mainly camera noise). Finally, the logarithm transform is employed to enhance the image contrast,

$$\begin{array}{c} p_{i}=\mathrm{lg}\left(p_{i}^{b}-p_{i}^{d}\right)-\mathrm{lg}\left(p_{i}^{o}-p_{i}^{d}\right) \end{array}$$

where $p_{i}^{b}$, $p_{i}^{d}$ and $p_{i}^{o}$ denote *i*-th column of background image, dark field image and projection image. The images are reconstructed by the classic FBP and the proposed iterative FBP, which are shown in Fig 8.

Since there is not a reference image, it is difficult to assess the image quality by some quantitative indexes. The difference between the real projection vectors and the reprojection vectors, i.e., *s*_1_ and *s*_2_ defined in Eq (9), are employed as the criterion. In this example, *s*_1_ = 299.4172, *s*_2_ = 93.4903.

## Discussion

As is shown in Fig 2 and Eq (5), the projection and reconstruction model are relatively simple in this paper. There are some factors are not considered, such as the quantization error (for example in *radon* function in Matlab, every pixel is divided into four sub-pixel and projects each sub-pixel separately) and projection noise. For such a model, the optimization of Eq (7) or Eq (8) will be rather complicated and difficult.

As previously shown in the flow chart (Fig 3), the computation burden induced by the iterative procedure mainly comes from the calculation of the additional reconstruction and filtering procedures. Obviously, the reconstruction part is the dominant portion. Generally, the number of iterative loops is unnecessary to be larger than four, which means the extra calculation burden is only twice or triple that of the classic FBP. To the best of our knowledge, the calculation burden is much less than that of the most IR algorithms, and is acceptable in general.

In the practical applications, since there are many factors that may degrade the quality of reconstructed image, such as the eccentricity of cylindrical axis and measurement noise, the image quality cannot be improved greatly. The eccentricity of cylindrical axis is the most serious factor for the iterative FBP algorithm because the distortion caused by the offset of projection data will be accumulated as the iterative procedure proceeds. The larger the eccentricity of cylindrical axis is, the less times the iterative procedure should be performed. According to our experience, for the experiment setups such as in SSRF, once iterative procedure is an appropriate choice.

## Conclusion

FBP algorithm is perfect for the continuous image model and scanning, however, the aliasing degradation cannot be avoided when the image and scanning model are discretized. Generally, there is a significant difference between the original projection data and the reprojection data using the classic FBP algorithm. Since the original projection data are the best information we have, a proper way is to take advantage of the original projection again to make the reprojection data approach the original projection data while improve reconstruction performance.

According to the analysis, a reprojection vector can be approximately expressed as the circular convolution of a certain kernel and a linear combination of the original projection vectors. The difference between the original projection and reprojection vector is served as the motivation to improve the reconstruction performance. It is filtered by a digital filter to compensate the difference caused by FBP, where the filter is designed to remove the filtering effect of the ramp filter. As a result, the sum of the reprojection vector and the filtered difference will approach the original projection vector. In the meantime, the filtered difference is performed inverse Radon transform and the result is added to the reconstruction image of last step to update it. This process can be repeated for several times until certain stopping criterion is satisfied. The results of some numerical simulations and practical applications demonstrate the proposed scheme have better superiority over the classic FBP algorithm in the term of MSE, UQI and MI. The results also show the proposed scheme have much better superiority over some iterative algorithms that depend only on the projection data, such as SIRT, SART and MAP-EM, in the term of reconstruction convergence speed and robustness.
